# Clinical and sociodemographic predictors of oral pain and eating problems among adult and senior Spaniards in the national survey performed in 2010

**DOI:** 10.4317/medoral.20400

**Published:** 2015-04-10

**Authors:** Javier Montero, Manuel Bravo, Antonio López-Valverde, Juan-Carlos Llodra

**Affiliations:** 1 PhD in Dentistry. Graduate in Odontology. Tenured Lecturer of Prosthodontics. Faculty of Medicine. University of Salamanca. Campus Miguel de Unamuno. PC: 37007. Spain; 2 PhD in Dentistry. Professor of Preventive and Community Dentistry. University of Granada. Faculty of Odontology. Campus de Cartuja PC 18071. Granada. Spain; 3 PhD in Dentistry. Associate Professor of Periodontics. University of Salamanca. Faculty of Medicine. University of Salamanca. Campus Miguel de Unamuno. PC: 37007. Spain; 4 PhD in Dentistry. Associate Professor of Preventive and Community Dentistry. University of Granada. Faculty of Odontology. Campus de Cartuja PC 18071. Granada. Spain

## Abstract

**Background:**

Pain and chewing difficulties have been identified as the strongest predictors of oral disadvantage. The aim of this study is to analyze and quantify the sociodemographic, behavioral and clinical factors modulating the oral pain and eating difficulties reported by Spanish adults and elderly Spanish people in the last National Oral Health Survey performed in 2010.

**Material and Methods:**

Data concerning pain and chewing difficulties were acquired on a Likert‑scale format from a representative sample of the Spanish general population with ages between 35-44 years (n=391) and 65‑74 years (n=405). Risk factors were identified using bivariate analysis, after which the crude association between risk factors (sociodemographic, behavioral and clinical) and outcome variables (pain and eating problems) was assessed by adjusted odds ratios, calculated by means of multivariate logistic regression.

**Results:**

Eating problems and oral pain were mainly associated with prosthetic and caries treatment needs as clinical predictors, but female sex was also seen to be a relevant and significant risk factor for suffering pain and eating restrictions. Paradoxically, after taking into account all the aforementioned predictors, the adults had an almost two‑fold higher risk of reporting pain or eating difficulties than the elderly subjects.

**Conclusions:**

In agreement with the results from the last national oral health survey, prosthetic and caries treatment needs should be considered key factors in determining the oral well‑being of the Spanish population. In sociodemographic terms, the women and adults were seen to be at a significantly higher risk of suffering pain and eating restrictions.

**Key words:** Oral pain, eating difficulties, oral health‑related quality of life, epidemiological studies, self‑assessment.

## Introduction

Data concerning the impact of dental conditions on oral health related quality of life (OHQoL) are of paramount importance because they can be used as a comparative measure, both transversal (between populations) and longitudinal (within populations), of oral health needs. In Spain, the results of the past five epidemiological studies performed between 1984 and 2010 revealed a gradual improvement in oral health (caries and periodontal disease) within all age groups, although mainly in the younger cohorts (1).

To complement the clinical data obtained from the national oral health surveys, data regarding the impact of oral conditions on the pain and the eating problems that individuals have had over the previous year were collected since the penultimate epidemiological study (2), and a secondary analysis performed on that database, demonstrated that 1 in 3 Spanish adults or seniors experienced oral pain as a result of oral disorders sometimes, or more frequently, in the previous year. In the same period, 1 in 5 Spanish adults and 1 in 4 Spanish elderly people had suffered from eating difficulties because of problems with their mouth, teeth, or dentures (2). After adjusting the regression model, it was concluded that caries and prosthetic treatment needs were the major factors in determining oral pain and eating difficulties among adult and elderly Spaniards. In addition, sex had an independent effect on the two outcomes, women being the most affected. However, since we performed a separate analysis for adults and the elderly, we were unable to assess the effect of age, although recent evidence from a large epidemiological study carried out in Australia suggests that the deleterious effect of dental disease is more pronounced in the young than in the old (3).

Accordingly, the aim of the present study is to replicate the secondary analysis but on the database of the last National Oral Health Survey (carried out in 2010) to quantify the effect of sociodemographic (sex and age), behavioral and clinical factors on the oral pain and eating difficulties reported by Spanish adults and elderly people.

## Material and Methods

The database used was generously lent by the Spanish Dental Association. A pathfinder epidemiological study was undertaken in Spain based on WHO recommendations for Oral Health Surveys published in 1997. This study was revised and approved by the Bioethics Committee of the Spanish Dental Association. Briefly, a representative clustered stratified sample of the general population with ages between ages 35 and 44 years (n=391) and 65 74 years (n=405) were recruited from 12 geographical areas during 2010 2011.

A consented standardized clinical oral examination for caries, periodontal disease, temporomandibular joint function (TMJ) and prosthodontic status was performed by calibrated examiners. TMJ disorders were only assessed in the case of the adults (n=391).

Data concerning the sociodemographic (age, sex, residence and social class) and behavioral (brushing habits) variables were recorded for all participants. Social class was categorized as high, medium high, medium, medium low and low, based upon the last employment of the head of the household (4). Furthermore all participants were questioned about how frequently they experienced any sort of pain (or eating difficulties) because of problems with their mouth, teeth or dentures in the previous 12 months. These two single items of subjective impairment were extracted from a European Project addressing Oral Health Subjective Indicators (5). The replies of the participants concerning pain or eating problems were recorded on a Likert type scale (0=never, 1=hardly ever, 2=sometimes, 3=fairly often and 4=very often). The prevalence of impact was estimated using the sometimes threshold to visualize the proportion of subjects suffering from pain or eating problems with a certain frequency. To analyze the subjective data, the total score was calculated by transforming the 0 4 range of the Likert scale into a 0 100 score, owing to the greater popularity of the percentage range and in order to achieve a better appreciation of the differences. Thus, 1 was recoded as 25; 2 as 50; 3 as 75, and 4 as 100, and only these values (0, 25, 50, 75 and 100) were used to summarize the impact level. Accordingly, the higher the total score, the greater the impact. This transformation has been used previously in an earlier study (2) but has also been validated in other symptom related instruments (6).

Means, standard deviations (SD) and confidence intervals (CI 95%) were used to describe the sample. Although a Likert type scale is an ordinal variable, the specific frequency range from “never” to “very often” has been widely used and has provided reliable results since its original development (7). We therefore considered that it could be used as a quantitative variable, providing a good picture of the prevalence of impact. Thus, we used parametric tests (Student´s t test and ANOVA) to compare the mean total score between two or more groups respectively, and the prevalence of impact was compared using the Chi-square Test. Modulating factors were initially explored using the Pear son and Spear man correlation coefficients (r). We performed a multivariate logistic regression analysis introducing by an stepwise forward selection method all the risk/protective factors observed in the bivariate analysis as the independent variables, and the presence of “pain” and “eating difficulties” (at the sometimes threshold) as the dependent variables.

The quantitative variables regarding caries were previously grouped into factors according to an exploratory factor analysis with varimax rotation in order to simplify the visualization of the modulating effect and to minimize the risk of removing important predictor variables due to collinearity. The Statistical Package for the Social Sciences v.20. (SPSS Inc., Chicago, IL) was used for the statistical analyses. The cut off level for statistical significance was 0.05.

## Results

Both groups studied, but mainly the youngest cohort, had a relative healthy oral status ( Table 1). Several clinical variables related to caries and periodontal diseases were found to be significantly different between the age cohorts. The DMFT (sum of decayed, missing and filled permanent teeth) index was mainly dependent on the number of filled teeth in the adults and missing teeth in the elderly subjects. The periodontal status and the level of attachment loss for most sextants were healthy in the adult cohort, but in the elderly individuals most of the sextants were missing and the most common periodontal status in the examinable sextants was coded as 2 (tartar and bleeding). Most adults did not carry a dental prosthesis (84.7%), although most of the seniors wore different types of prosthesis (mainly complete or partial dentures). Most of the subjects had no prosthetic needs (66.2%), but most of the elderly were in need of multiple tooth replacements (47.2%).  Table 1 also summarizes the sociodemographic and behavioral profile of both age cohorts. Sex was equally distributed between the cohorts, but regarding Social Class, the medium low category was significantly more common in the elderly than in the adult individuals. Brushing habits were significantly better within the younger cohort. The place of residence had a similar distribution within each age cohort.

**Table 1:**
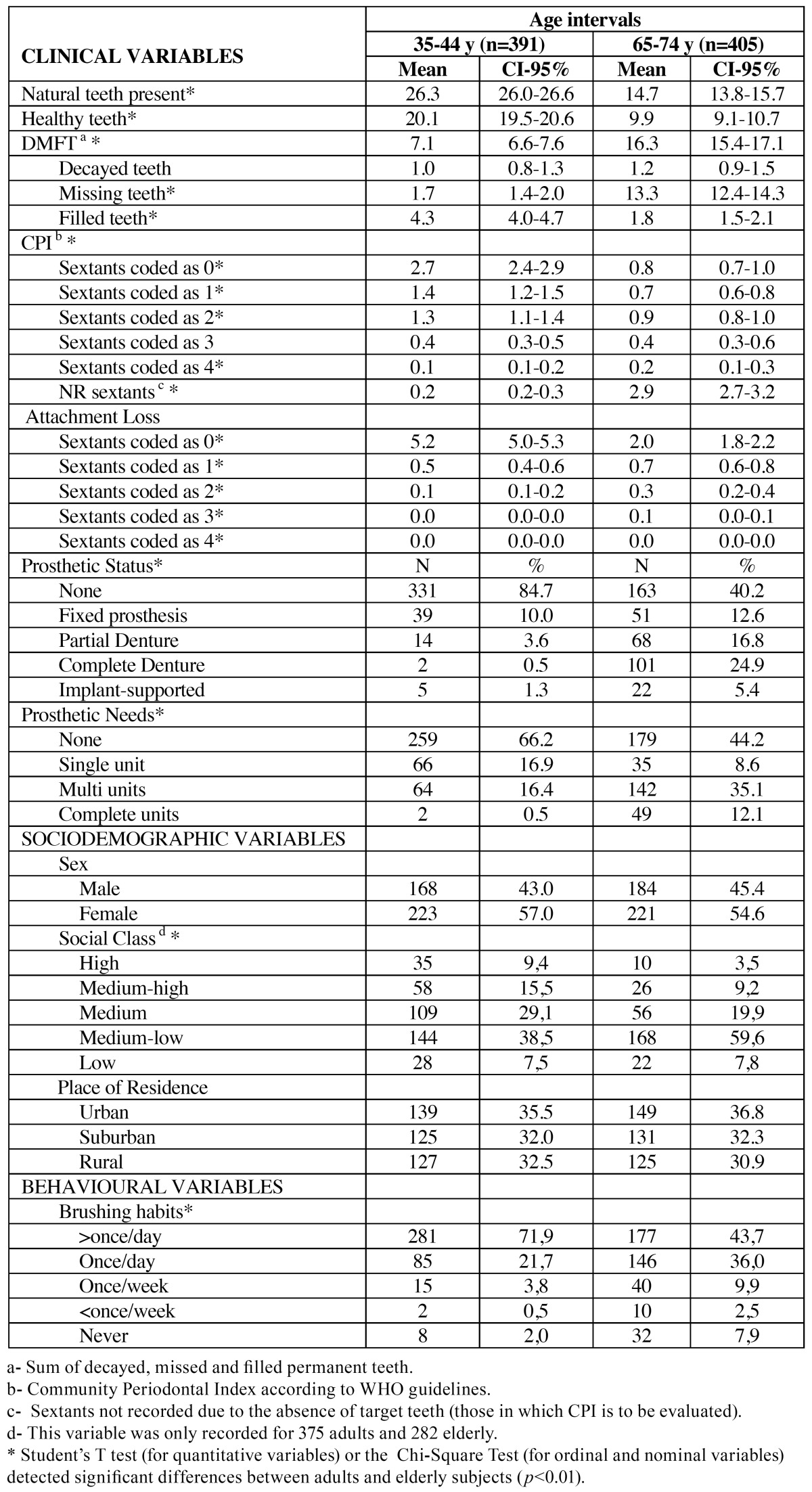
Clinical, behavioural and sociodemographic description of age cohorts (n=796).

The prevalence of pain and eating problems in the whole sample (using the sometimes threshold) were 25.1% and 17.5% respectively ( Table 2). Using the fairly often threshold the prevalence of pain and eating problems were 6.7% and 6.2% respectively. The prevalence of pain and eating problems were similar in both cohorts, but the total eating score was significantly higher in the elderly people than in the adults. Pain and eating difficulties were also influenced by social class; i.e. the lower the class, the higher impact (this trend was clearer for eating problems than for oral pain). The women also showed a higher level and prevalence of pain and eating problems. Brushing habits were also associated in the expected direction with prevalence and the scores of oral impact. Furthermore, rural residents reported a higher impact of pain and eating problems than their urban and suburban counterparts.

**Table 2:**
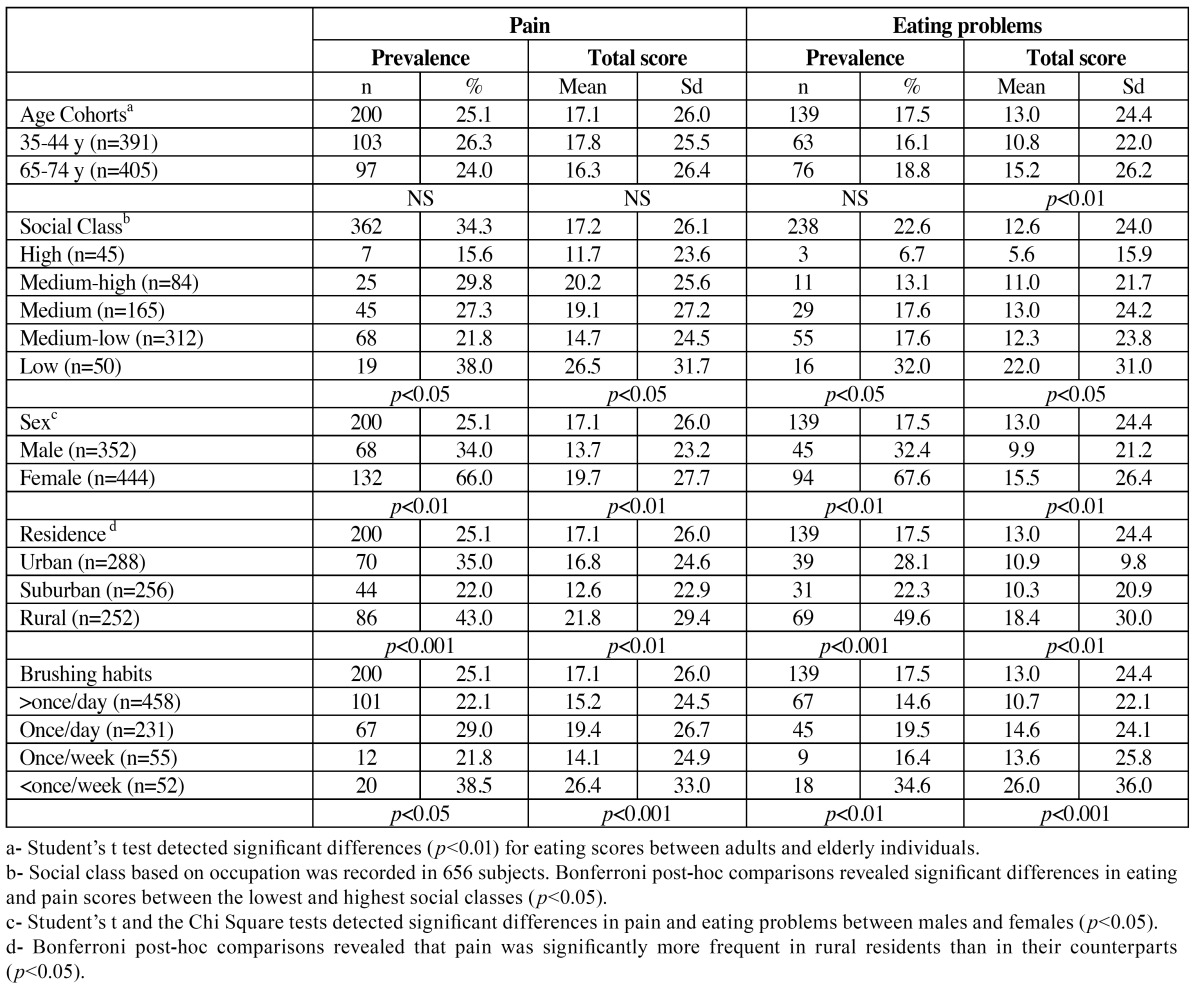
Prevalence and total scores of pain and eating problems compared with selected sociodemographic and behavioural characteristics of the respondents (n=1080).

The prevalence of oral pain and eating problems was significantly higher in subjects with TMJ symptoms than their counterparts ( Table 3). Eating problems were significantly more prevalent among the subjects with TMJ pain after palpation than their counterparts, although this expected effect on the prevalence of oral pain did not reach the level of significance (Chi: 1.71; df=1, *p*=0.19). Prosthetic needs were significantly related to the prevalence and level of impact of the pain and eating problems in a coherent way. Nevertheless, periodontal needs were only related to the prevalence and level of oral pain but not to eating problems. Those with no periodontal needs or who merely needed to improve their brushing habits had a significantly lower prevalence and frequency of oral pain than that reported by the rest of the periodontal subgroups. The number of healthy, missing, or decayed teeth was also found to modulate the prevalence and level of both subjective impairments, but above all eating difficulties. In this sample, the total scores for pain and eating problems were significantly correlated (r=0.59; *p*<0.05). Some sociodemographic, behavioral and clinical modulating factors were observed, but the main clinical factor associated with pain was the number of teeth needing fillings (r=0.19; *p*<0.01) or endodontic treatment (r=0.25; *p*<0.01). Eating problems were mainly correlated with prosthodontic needs (r=0.23; *p*<0.01), but also with the number of natural teeth present (r=-0.17; *p*<0.01), missing teeth (r=0.17; *p*<0.01), and the DMFT index (r=0.17; *p*<0.01). Age, social class and brushing habits demonstrated significant but weak correlations with both pain or eating problems, ranging from r=0.08 to r=0.12.

**Table 3:**
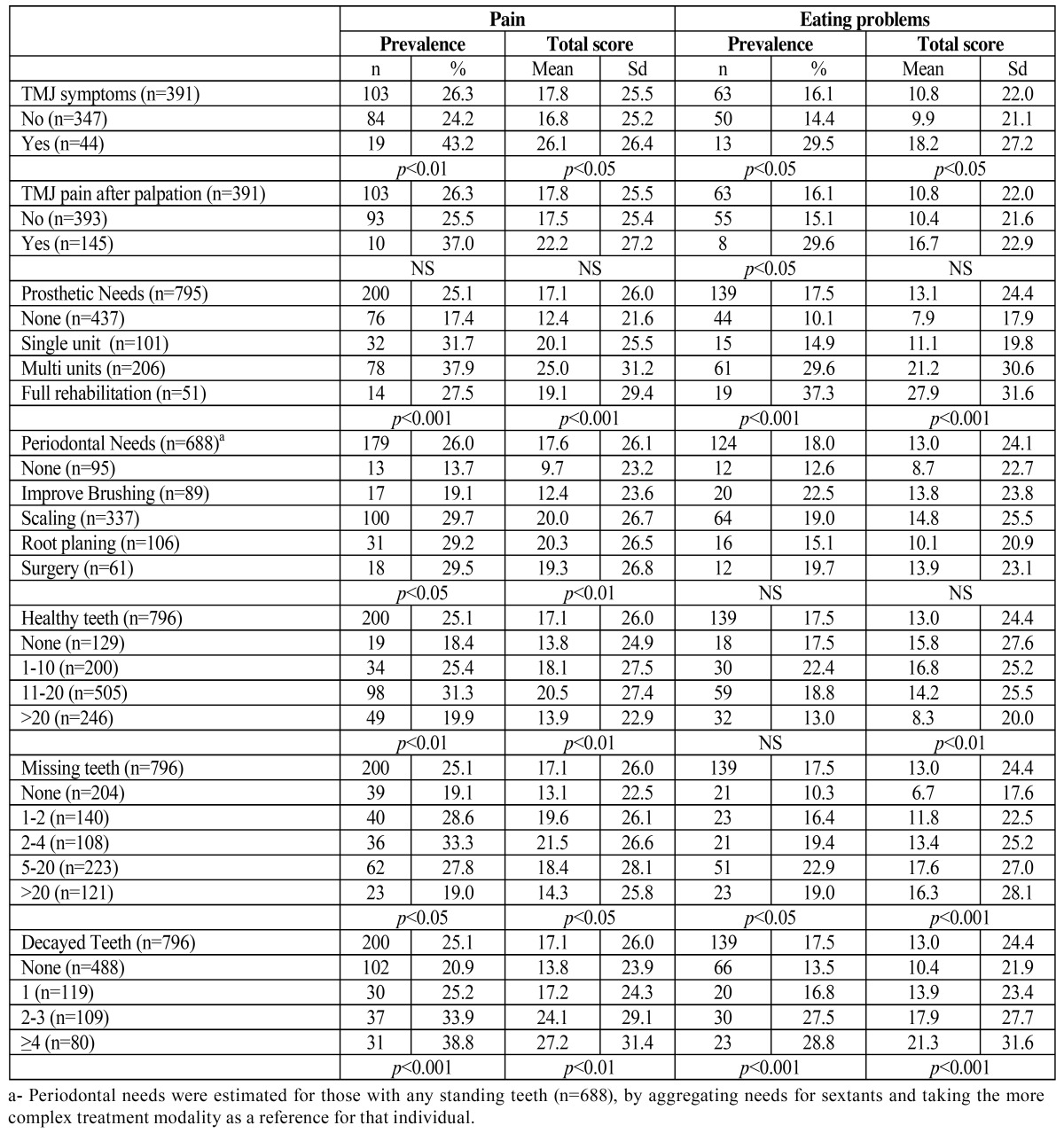
Prevalence and total scores of pain and eating problems in some clinical conditions.

Once the main clinical factors associated with pain and eating problems had been determined, a principal component analysis was performed on the most relevant quantitative variables (r>0.15) in order to reduce the number of these variables and to capture the underlying clinical domains for both cohorts before applying the logistic regression analysis. According to principal component analysis, eight clinical variables proved to be coherently loaded within two factors (designated Missing Factor and Caries Factor). The Missing Factor was conceived as a latent variable related to tooth loss, as suggested by the nature and factor loadings of the variables integrating it. Caries Factor comprised decayed teeth and those in need of treatment. Both factors, with eigenvalues above 2, explained more than 75% of the variance and were included in the subsequent logistic regressions analysis.

 Table 4 shows the results of the logistic regression for predicting pain and eating problems among the Spanish general population. In general, the Odds Ratios were strongly reduced after adjustment in both effects (pain and eating restrictions) and after this process several independent variables were seen to be non significant, as was the case of social class, place of residence, brushing habits, and periodontal needs; however, none of them changed the direction of the association revealed in the bivariate analysis.

**Table 4:**
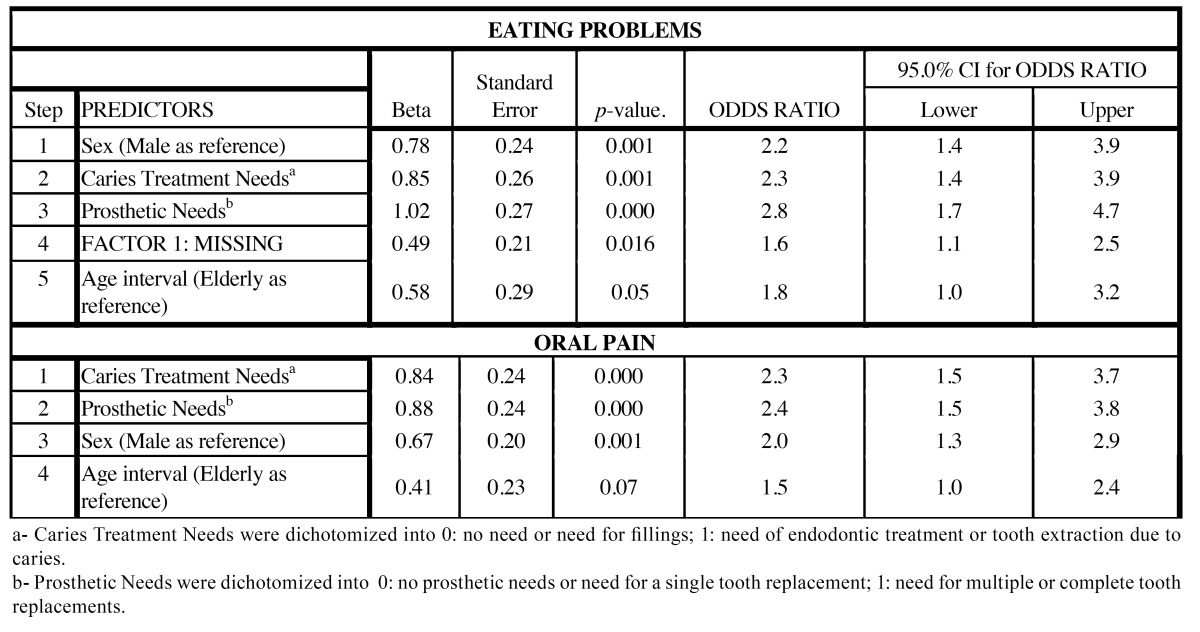
Logistic Regression Models using a stepwise selection method for predicting eating problems and dental pain in Spanish adults (n=391) and elderly people (n=405).

Pain and eating problems were mainly modulated by prosthetic treatment needs, caries treatment needs, sex and age. The Missing Factor was a significant predictor for eating problems but not for pain. According to this model, individuals needing multiple or total tooth replacements have 2.8 –fold greater risk (OR) of suffering from eating problems and a 2.4-fold higher risk of reporting pain than those with no prosthetic needs or needs for single replacements. Similarly, those needing endodontic treatment or tooth extraction due to caries have a 2.3-fold greater risk (OR CI 95%: 1.4 3.9) of suffering from eating problems and pain (OR=2.3, IC 95%: 1.5 3.7) than those without such treatment needs. Moreover, women are at higher risk than men of suffering from eating problems (OR CI 95%: 1.4 3.9) and also oral pain (OR CI 95%: 1.3 2.9). The presence of chewing difficulties was also significantly associated with the Missing Factor. In contrast to what was observed in the bivariate analysis, after controlling all the aforementioned predictors the adults had an almost two fold higher risk of reporting pain or eating difficulties than the elderly subjects (note that for oral pain this predictor was significant at a *p*-value of 0.07).

## Discussion

Pain and chewing difficulties are the two most common outcomes of oral illness, such as caries, periodontal diseases or edentulism, and they tend to be prevalent among the adult and elderly populations. Accordingly, pain and eating problems are the two strongest predictors of oral disadvantage (8) and the most relevant domains of the OHQoL construct (9). Both dimensions were collected as single item instruments, in order to ease the application in this national survey. It should be noted that in the present study sample size was about 25% smaller than in the earlier study (2), (which comprised 540 subjects in each age group), but the sociodemographic profile (sex and social class) was similar. Brushing habits seem to have improved, but only within the adult cohort, in which 15% more adults brushed their teeth more than once a day. In clinical terms, the dental status observed here is healthier than that found in the previous study (regarding caries and periodontal disease) in both age cohorts. Nevertheless, the prosthetic needs in our setting among the adults (33.8%) and elderly (55.8%) were greater than in the previous survey (i.e. 27.8% and 34.8% respectively), this increase being due to need for single dental replacement in the adult cohort and for multiple/full replacements in the elderly. By contrast, in general the prevalence of self reported oral pain or eating problems has been reduced by about 10% and 5% respectively, this reduction being slightly greater in the case of the elderly subjects. If in the previous study we reported that 1 in 3 Spanish people reported experiencing oral pain as a result of oral disorders in the previous year, we have now found 1 in 4 ( Table 2). In the same sense, the prevalence of individuals’ eating difficulties due to problems with their mouth, teeth, or dentures has also been reduced from 1 in 4 to 1 in 5. This reduction may be a consequence of the better oral health status (as regards caries and periodontal disease), but could also be influenced by several non clinical factors, such as an increase in people’s resilience when faced with problems deriving from the economic crisis. Financial hardship may force the acquisition of a sense of resilience, which would increase the capacity for bearing such edentulism related impairments.

In comparison with other countries, the prevalence of pain and eating problems reported here was comparable to that reported by other authors who used the same questions at the same threshold (sometimes) for adults in Australia (25.6% in pain and 31.3% in eating), in the United States (19.5% pain and 16.8% for eating) (10) and in the United Kingdom (11). However, on using the fairly often threshold these prevalences were slightly higher than that reported for Swedish adults and elderly people (3% in pain, 4% in for eating) (12), Canadian adults (13) (5.4 for pain and 3.9% for eating problems) and Thai adults (14) (10.6% for pain and 15.8% for chewing). As expected, we observed that the healthier the oral status, the lower the prevalence of pain and eating problems within the same reference population. We believe that the latent burden of impact observed here may be related to the burden of untreated dental disease owing to fear or economic barriers (15). A regular dental visiting pattern has proved to be related to both the oral health status and the OHQoL among adult and elderly Spaniards (16). Another worrying finding is that the prevalence of pain and eating restrictions is still much higher in the low social class respondents (than in individuals from higher social classes ( Table 2). It has been suggested that socio economic conditions might influence OHRQoL both directly and indirectly (17,18). However, the level of tolerance/endurance to oral disadvantage by this subgroup accustomed to long-lasting oral disturbances could be the underlying reason for such a paradoxical result (16). According to the logistic regression model, social class was not significant but age seemed to modulate both perceptions, younger individuals being at higher risk of suffering from pain or eating problems ( Table 4).

Some clinical conditions were found to be related to the prevalence and the total scores of pain and chewing difficulties (see  Table 3). In agreement with previous results (2), TMJ disorders were mainly related to eating problems. The impact of TMJ disorders on quality of life has been reported previously (19,20) and our data suggest that although such disorders are rare among the general adult population, subjects with TMJ symptoms report significantly higher impacts than those without pain. (See  Table 3). Dental treatment needs (caries, periodontal and prosthesis) were significantly related to pain and eating problems in the expected direction. In agreement with the reference study (2), we found a clear gradient in prevalence rates and the total scores of both items according to the prosthodontics, periodontal and caries needs, using the same categorization strategy as depicted in  Table 3. However, periodontal status and periodontal needs are not able to predict pain or chewing difficulties when a multivariate analysis is performed ( Table 4). We are well aware that the most advanced states of periodontal disease clearly impinge on oral well being, as reported elsewhere (21,22), but our findings come from general population based studies, in which periodontal disease is a very common but usually not very severe condition, and thus bearable in terms of oral function.

Nevertheless, the needs for caries treatment proved to be an important modulating factor that impinges on both the pain and the eating difficulties of the Spanish population. Since the above mentioned findings had already been observed previously, the Caries Factor could be proposed as one of the most relevant modulating factors, above all for oral pain and mainly when an invasive treatment is needed to resolve it (23).

Regarding prosthodontic needs, it should be noted that the higher number of missing teeth, the greater the impact on eating. In addition, missing teeth also seem to be proportionally correlated with the pain experienced by subjects whose missing teeth have not been replaced by a dental prosthesis ( Table 3). In concordance with the results of a recent meta analysis (24), prosthetic needs have been shown to be the most relevant factor affecting chewing in Spanish adults and elderly people after the logistic model has been adjusted ( Table 4). In fact, caries and the prosthetic needs could be the key factors accounting for the discrepant prevalence of impact on eating among age cohorts, social classes and places of residence.

However, the differences found between males and females do not seem to be related to oral health status but, instead, to certain particularities in the conception of oral well being, and perhaps some personality traits (25), leading women to perceive a greater disadvantage and less satisfaction than males in comparable clinical situations. This factor has been widely broached in epidemiological studies (2,1-14,26,27).

In sum, in this model, after controlling for the interference of the confounding factors, two key clinical factors (prosthetic and caries treatment needs) and two key sociodemographic factors (sex and age) emerge. The impact generated by the loss of teeth and prosthetic needs has been addressed by several authors (14,27-29), reporting its modulating effect. According to the results of our study, it may be confirmed that oral health problems are reduced with age when all the confounding factors are controlled in a multivariate regression model. It should be noted that this association proved to be insignificant or even inversely related in the bivariate analyses ( Table 2). This is perplexing because dental disease is chronic and cumulative, gradually leading to tooth loss, and hence occurs more frequently in older than in younger subjects. The paradoxical association between age and subjective oral health, which contradicts standardized assumptions, has been discussed in depth elsewhere (3). It is feasible that this age related effect could be cohort dependent, as reported by other authors for Australian and British people (11). The signs and symptoms of oral disease seem to be more deleterious to subjective oral health when they occur early on in adulthood than when they occur in old age (3). We believe that since elderly people have spent more time coping with the consequences of edentulism they have gradually adapted to the functional changes required or imposed by this long lasting condition, which otherwise is conceived as a normal consequence of aging. This latter issue would be what really impinges on the perception of the younger cohort. The findings of the present study suggest that clinical and sociodemographic factors have independent effects on the presence of pain and eating problems.
